# Management of a Mycotic Aneurysm in a Patient with COVID-19: A Case Report

**DOI:** 10.3390/medicina57060620

**Published:** 2021-06-14

**Authors:** Muzammil H. Syed, Mark Wheatcroft, Danny Marcuzzi, Hooman Hennessey, Mohammad Qadura

**Affiliations:** 1 Division of Vascular Surgery, St. Michael’s Hospital, Toronto, ON M5B 1W8, Canada; muzammil.syed@mail.utoronto.ca (M.H.S.); mark.wheatcroft@unityhealth.to (M.W.); 2 Department of Surgery, University of Toronto, Toronto, ON M5B 1W8, Canada; 3 Department of Diagnostic Imaging, St. Michael’s Hospital, Toronto, ON M5B 1W8, Canada; Danny.Marcuzzi@unityhealth.to; 4 Division of Vascular and Interventional Radiology, Health Sciences North, Northern Ontario School of Medicine, Sudbury, ON M5B 1W8, Canada; hhennessey@nosm.ca; 5 Keenan Research Centre for Biomedical Science, Li Ka Shing Knowledge Institute of St. Michael’s Hospital, Toronto, ON M5B 1W8, Canada

**Keywords:** mycotic aneurysm, COVID-19, endovascular stents, rifampin, Klebsiella pneumoniae, endoleak, follow-up studies, SARS-CoV-2

## Abstract

The aim of this paper is to share our experience in managing a patient with Klebsiella pneumoniae mycotic abdominal aortic aneurysm who was also infected with COVID-19. A 69-year-old male was transferred to our hospital for the management of an infra-renal mycotic abdominal aortic aneurysm. During his hospital course, the patient contracted severe acute respiratory syndrome coronavirus 2 (SARS-CoV-2). He was intubated due to respiratory distress. Over a short period, his mycotic aneurysm increased in size from 2.5 cm to 3.9 cm. An emergency repair of his expanding aneurysm was achieved using our previously described protocol of coating endovascular stents with rifampin. The patient was managed with a rifampin-coated endovascular stent graft without any major complications. Postoperatively, the patient did not demonstrate any neurological deficits nor any vascular compromise. He remained afebrile during his postoperative course and was extubated sometime thereafter. He was then transferred to the ward for additional monitoring prior to his discharge to a rehab hospital while being on long-term antibiotics. During his hospital stay, he was monitored with serial ultrasounds to ensure the absence of abscess formation, aortic aneurysm growth or graft endoleak. At 6 weeks after stent graft placement, he underwent a CT scan, which showed a patent stent graft, with a residual sac size of 2.5 cm without any evidence of abscess or endoleak. Over a follow-up period of 180 days, the patient remained asymptomatic while remaining on long-term antibiotics. Thus, in patients whose surgical risk is prohibitive, endovascular stent grafts can be used as a bridge to definitive surgical management.

## 1. Introduction

Since December 2019, reports started emerging from China describing cases of patients with peculiar respiratory complications arising from an unknown cause [1]. This virus was later called severe acute respiratory syndrome coronavirus 2 (SARS-CoV-2) that causes a disease called COVID-19 (coronavirus) [2]. At the time of writing this manuscript, over 173 million cases of COVID-19 and 3.5 million deaths had been reported globally (as per the COVID-19 online dashboard developed by the Center for Systems Science and Engineering at Johns Hopkins University) [3]. The management of COVID-19 patients, in their sickest form, is complex as they can remain intubated and ventilated in a critically ill state for weeks, if not longer. As such, these patients are at risk of developing numerous other serious complications during their hospitalization, which must be concurrently diagnosed and treated. Triaging how best to approach each of these issues in the context of the patient’s other life-threatening illnesses is done by carefully balancing the patient’s clinical state and the risks and benefits of each procedure. Despite a low incidence, mycotic aneurysms are associated with high morbidity and mortality [4,5]. Patients usually present with the triad of fever, abdominal pain, and pulsatile abdominal mass. In addition to medical management with antimicrobials, the standard of care is definitive repair via a standard open surgical approach, which involves surgical resection, debridement, and revascularization [6,7]. However, such a surgical approach poses a significant surgical and medical burden on the patients. Therefore, for patients who are unlikely to withstand major open surgery, the use of endovascular stent grafts has been proposed as a bridge treatment (i.e., a temporary treatment measure taken until open revascularization can be performed) [8]. In this case report, we describe the management and follow-up of a patient with a rapidly expanding mycotic aneurysm who concurrently developed COVID-19 requiring intubation and ventilatory support. To the best of our knowledge, no case report has previously documented the management of a mycotic aneurysm in patients positive for COVID-19. The patient consented and agreed to have their case details and any associated images published. 

## 2. Case Report

**Figure 1:**
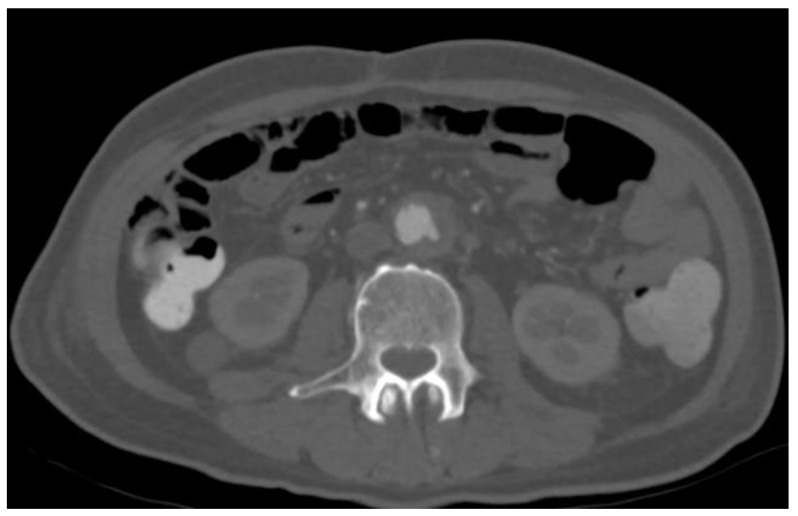
Interim development of an infra-renal mycotic aneurysm measuring 2.5 cm.

A 69-year-old man with a prior history of coronary artery disease, diabetes, hypertension, and intraductal papillary mucinous neoplasm (IPMN) resected with a Whipple’s procedure in 2000 presented to an outside institution with symptoms consistent with septic shock. He was hemodynamically unstable with hypotension, tachycardia, and tachypnea. His laboratory work-up and imaging demonstrated Klebsiella pneumoniae bacteremia, which was further complicated by a pyogenic liver abscess. He was managed with intravenous antibiotics as well as percutaneous drainage of the liver abscess. Thereafter, his hospital course was uneventful, and he was discharged home on oral antibiotics. The patient returned to the emergency department 15 days later with significant abdominal pain. A repeat CT scan showed the resolution of the liver abscess, but there was now the presence of an infra-renal multi-lobed aortic dilatation measuring 2.5 cm in maximal diameter (Figure 1). Thus, given the patient’s clinical features and radiographical findings of aortic dilatation, a mycotic aneurysm was highly suspected.

At that point, the patient’s antibiotics were broadened empirically, and he was transferred to our hospital for definitive management. On presentation, the patient’s vital signs were stable, but he complained of mild abdominal pain that was controlled with medication. His bloodwork was unremarkable, with negative blood cultures. However, given his previous history of bacteremia and the rapid development of a multi-lobar aneurysm that is highly suspicious of a mycotic aneurysm, he remained on intravenous antibiotics.

**Figure 2:**
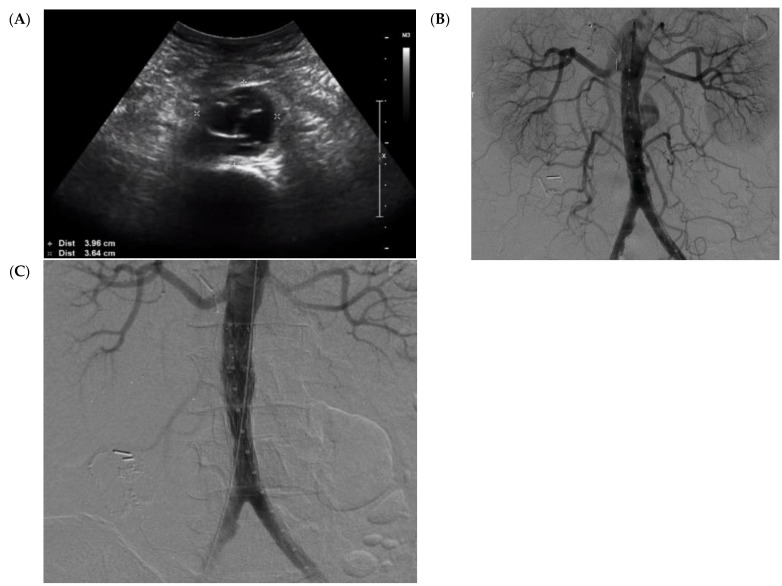
(**A**) Preoperative duplex ultrasound demonstrating rapid expansion of an infra-renal mycotic aortic aneurysm to a maximum diameter of 3.96 cm. (**B**) Anterior posterior intraoperative angiogram demonstrating a mycotic aneurysm. (**C**) Final intraoperative angiogram demonstrating a sealed mycotic aneurysm using an endovascular stent graft.

As the patient was stable and did not have any contraindications for major arterial surgery, we planned to repair his mycotic aneurysm through an open surgical approach. Unfortunately, the patient developed respiratory symptoms while in hospital. After extensive testing, it was confirmed that the patient had an active case of COVID-19 infection. The patient’s respiratory status deteriorated to the point of requiring intubation and mechanical ventilation. Meanwhile, repeat ultrasound at day 7 (since admission to our hospital) showed expansion of his mycotic aortic aneurysm to a maximum diameter of 3.96 cm, with an affected length of 3.8 cm (Figure 2A). Given the deterioration in his medical status as well as the rapid expansion of his aneurysm, a minimally invasive endovascular procedure was preferred over open repair. In an effort to reduce the risk of graft infection, we planned for the endovascular stent graft to be coated with rifampin, as described previously [9]. Rifampin-impregnated grafts have been described to minimize the risk of prostatic graft infections [10,11,12].

**Figure 3:**
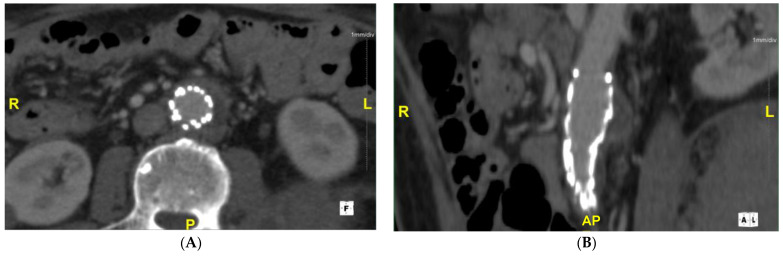
CT scan (**A**) axial view and (**B**) coronal view at 6 weeks after endovascular repair, demonstrating a stable sac without any noticeable evidence of mycotic aneurysm progression or endoleak. R = right; L = Left; P = Posterior, AP = Anterior Posterior.

The patient was managed at our hybrid operative room. In Figure 2B, the initial angiogram (under fluoroscopic guidance with a percutaneous approach) confirms the presence of an infra-renal mycotic aneurysm. To manage this aneurysm, two rifampin-pre-soaked Cook^®®^ endovascular graft main-body extension grafts were deployed within the aorta to seal the mycotic aneurysm. Throughout the procedure, the patient was anticoagulated with heparin with an activated clotting time (ACT) of >250 s to reduce possible thrombotic complications associated with COVID-19. Intraoperative imaging showed the exclusion of the mycotic aneurysm and a patent stent graft (Figure 2C). The patient was then transferred to the intensive care unit for additional monitoring and continued management of his respiratory status and COVID-19 infection. He remained on intravenous antibiotics throughout his hospital stay, with negative blood cultures. A week later, the patient was extubated. Serial aortic ultrasounds were completed on weeks 1, 2, 3, and 4 postoperatively, which demonstrated a patent stent graft with no obvious abscess formation or endoleak. Once the patient was cleared to be medically discharged, he was transferred to a rehabilitation center while remaining on intravenous ceftriaxone. He was monitored closely at our ambulatory clinic with serial imaging. At postoperative week 6, repeat CT scanning was done to assess his stent graft. His images continued to demonstrate no obvious aortic wall enhancement, abscess formation, or endoleak, as well as a stable residual aortic sac of 2.6 cm (Figure 3). 

**Figure 4:**
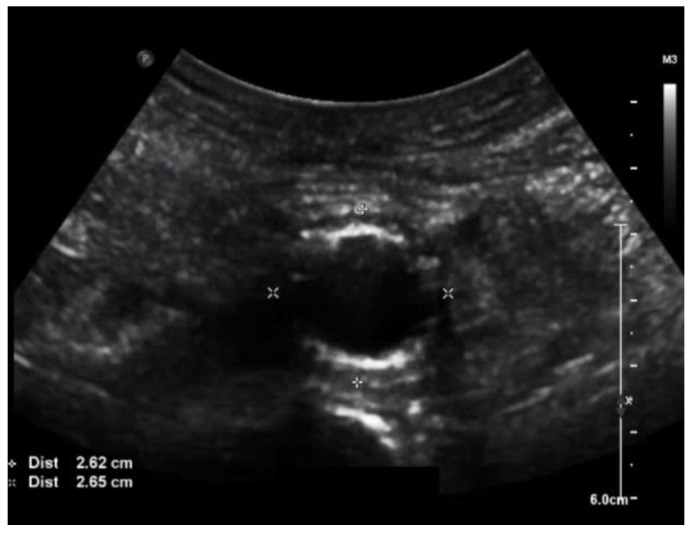
Duplex ultrasound at 6 months after endovascular repair of the mycotic aneurysm, demonstrating a stable sac that had decreased to a size of 2.65 cm.

The patient was seen last at our ambulatory clinic at the 6-month follow-up milestone since his aortic surgery. His aortic ultrasound once again showed no obvious abscess formation or endoleak, with a stable residual aortic sac (Figure 4). The residual sac size on his last ultrasound was 2.65 cm. Since the patient has remained stable on long-term oral antibiotics, and his stent graft is patent with a stable residual aortic sac, we have opted to continue surveillance and delay his open aortic repair to a later time.

## 3. Discussion

Mycotic aneurysms were first described by Sir William Osler in 1885. This term was used to describe an infected aorta secondary to complications from endocarditis [13]. However, this term has been adopted by many physicians while referring to infected aneurysms. Although the incidence of mycotic aneurysms is low, estimated to be 0.6% to 2% of all aortic aneurysms [14], the morbidity and mortality associated with this condition is high [5]. In the absence of definitive surgical intervention, death from arterial rupture is the main cause of mortality. The gold standard management of mycotic aneurysms involves early initiation of antibiotic therapy in conjunction with surgical debridement of the infected aorta, in addition to arterial revascularization. However, when a patient’s clinical condition is prohibitive to immediate definitive surgical management, other solutions must be entertained.

Therefore, a minimally invasive approach such as the placement of endovascular stent grafts has been used to temporarily seal the mycotic aneurysm while patients are hemodynamically unstable or otherwise too sick to undergo definitive surgical repair. To try to mitigate against re-infection of new implants, our group and others have previously described an algorithm that can guide surgeons on coating endovascular stent grafts with rifampin [9]. In the postoperative course, along with long-term antibiotics, patients are closely monitored with imaging surveillance assessing graft patency and any signs of local re-infection. Through the use of such a minimally invasive technique, patients might have the time to stabilize prior to receiving the definitive surgical repair. 

In the context of an unwell patient, temporizing the aneurysm with an endovascular stent graft seems to be a reasonable alternative [15]. In a large retrospective multi-centered study [4], Sörelius et al. reported 123 patients with mycotic aortic aneurysms that were treated with an endovascular approach, 6 of which were converted to an open repair during the mean follow-up of 35 months. The survival rate at 10 years was 41%, and infection-related mortality occurred in 23 patients, of which 9 occurred after the discontinuation of antibiotic therapy. The authors concluded that the endovascular treatment of mycotic aortic aneurysms is durable and feasible for most patients who are otherwise unfit for open repair [4]. 

In a case series by Sörelius et al., endovascular stent grafting was used to treat 11 patients [16]. The authors concluded that endovascular repair for mycotic aneurysms is feasible, with acceptable morbidity and mortality rates, provided that long-term antibiotics are continued and close follow-up is ensured, and definitive open repair is later found necessary in selected cases. In another case series by Lew et al. [17], the authors demonstrated a relatively low mortality rate of 22% while managing mycotic aortic aneurysms with endovascular stent grafts. The authors suggest the use of endovascular stent grafts as a bridge to open surgical repair in unstable patients with mycotic aortic aneurysms. 

In a large Swedish study on management of mycotic thoracic aortic aneurysms [18], the authors described the use of endovascular stent grafts in managing 52 patients with mycotic aortic aneurysms. At five years, the authors reported a survival rate of 71%, a re-infection rate of 17%, and a re-operation rate of 17%. Therefore, they concluded that endovascular stent grafting has acceptable long- and short-term survival rates. Lastly, Puppala et al. [19] reported 34 patients with a 100% technical success rate who were treated with endovascular stent grafts for mycotic aortic aneurysms. Over a 30-day follow-up period, the authors reported re-operation procedures in four patients, while two mortalities were recorded. Although this was a short-term study, the authors supported the use of minimally invasive endovascular procedures over open surgery in clinically unstable patients who are otherwise unfit for open repair. 

In contrast to the aforementioned studies, we were able to successfully deploy an endovascular, rifampin-coated stent in a patient with a concurrent mycotic aneurysm and COVID-19—something that has not been reported previously. We opted for an endovascular approach to manage this patient’s rapidly expanding mycotic aneurysm since he was clinically unstable and not fit for open repair. We used our previously described method of coating endovascular stents with rifampin [9]. Over the follow-up period of six months, the patient’s mycotic aneurysmal sac remained stable, and we are pleased to report that our endovascular approach has allowed the patient the time to overcome his COVID-19 infection. 

## 4. Conclusions

During the COVID-19 pandemic, we will continue to care for patients with vascular emergencies requiring prompt medical attention and treatment. In unstable patients, especially with the COVID-19 pandemic, minimally invasive procedures are preferred over open interventions. In patients with mycotic aneurysms and concomitant symptomatic COVID-19 infection, when possible and if anatomically acceptable, we recommend the use of minimally invasive procedures such as rifampin-soaked endovascular stent grafts placed percutaneously.

## Data Availability

Not applicable.
